# Survival after pulmonary metastasectomy for pancreatobiliary carcinomas: insights from a nationwide multicenter database

**DOI:** 10.1007/s00595-026-03389-8

**Published:** 2026-07-20

**Authors:** Taisuke Kaiho, Takahide Toyoda, Hidemi Suzuki, Yoshikane Yamauchi, Noriaki Sakakura, Mingyon Mun, Hiroshi Hashimoto, Takekazu Iwata, Yasushi Shintani, Masayuki Chida, Haruhisa Matsuguma, Keisuke Asakura, Makoto Endo, Tomohiko Iida, Mitsutomo Kohno, Haruhiko Kondo, Norihiko Ikeda, Masaaki Sato, Yukinori Sakao

**Affiliations:** 1 Department of General Thoracic Surgery, Chiba University Graduate School of Medicine, 1-8-1, Inohana, Chuo-ku, Chiba, 260-8670 Japan; 2 Department of Surgery, Teikyo University School of Medicine, Tokyo, Japan; 3 Department of Thoracic Surgery, Aichi Cancer Center, Aichi, Japan; 4 Department of Thoracic Surgical Oncology, The Cancer Institute Hospital, Japanese Foundation for Cancer Research, Tokyo, Japan; 5 Department of Thoracic Surgery, National Defense Medical College, Saitama, Japan; 6 Division of Thoracic Surgery, Chiba Cancer Center, Chiba, Japan; 7 Department of General Thoracic Surgery, Osaka University Graduate School of Medicine, Osaka, Japan; 8 Department of General Thoracic Surgery, Dokkyo Medical University, Tochigi, Japan; 9 Division of Thoracic Surgery, Tochigi Cancer Center, Tochigi, Japan; 10 Division of Thoracic Surgery, Department of Surgery, Keio University School of Medicine, Tokyo, Japan; 11 Department of Thoracic Surgery, Yamagata Prefectural Central Hospital, Yamagata, Japan; 12 Department of Thoracic Surgery, Kimitsu Chuo Hospital, Chiba, Japan; 13 Department of General Thoracic Surgery, Saitama Medical Center, Saitama Medical University, Kawagoe, Japan; 14 Division of Thoracic and Thyroid Surgery, Kyorin University, Tokyo, Japan; 15 Division of Thoracic and Thyroid Surgery, Tokyo Medical University, Tokyo, Japan; 16 Department of Thoracic Surgery, The University of Tokyo Graduate School of Medicine, Tokyo, Japan

**Keywords:** Surgery, Pulmonary metastasis, Pancreatobiliary carcinomas, Pancreatic carcinomas, Biliary tract carcinomas

## Abstract

**Purpose:**

Pancreatic carcinoma (PC) and biliary tract carcinoma (BTC) may metastasize to the lungs, and surgical resection of these metastases should be considered in selected patients with controlled primary disease. This study evaluated the survival outcomes and prognostic factors in patients who underwent pulmonary metastasectomy for pancreatobiliary carcinoma.

**Methods:**

Seventy-eight patients who underwent lung metastasectomy between 1993 and 2019 were included in the Metastatic Lung Tumor Study Group of the Japan database. The clinicopathological characteristics including overall survival (OS), pulmonary metastasis-free survival (PmFS), and disease-free interval (DFI) from primary surgery to lung metastasectomy were analyzed.

**Results:**

We included 41 men and 37 women with a mean age of 67 years. The primary cancers included 56 PC and 22 BTC cases. Partial resection was performed in 52 patients, segmentectomy in 16, and lobectomy in 10. Five-year OS was 38.3% (43.2% for PC and 28.1% for BTC). Five-year PmFS was 28.3% (28.4% for PC and 27.7% for BTC). The median DFI was 36.0 months (21.0–47.5 months, interquartile range). Larger metastases (> 2 cm) were significantly associated with a poorer survival (hazard ratio, 2.03; 95% confidence interval, 1.06–3.90, *p* = 0.03).

**Conclusion:**

Lung metastasectomy may be a viable treatment option for select patients with pancreatobiliary carcinoma, particularly those with smaller metastases.

**Supplementary Information:**

The online version contains supplementary material available at https://doi.org/10.1007/s00595-026-03389-8.

## Introduction

Pancreatobiliary carcinomas are characterized by a highly aggressive biological behavior and poor survival outcomes [1, 2]. Given that pancreatic and biliary tract carcinomas share several clinical challenges, such as locally advanced disease, frequent metastasis, a poor prognosis, and complex surgical management, they are often analyzed together in clinical studies [3–6]. Despite advances in surgical techniques, interventional therapies, and chemotherapy, long-term prognosis remains dismal, largely due to high recurrence rates, even after complete surgical resection. Among recurrent sites, the lungs are a relatively common location for the first recurrence, and in selected cases, recurrence may be limited to solitary pulmonary lesions [7]. Pulmonary metastasis frequently occurs as part of a widespread metastatic disease involving multiple organs. Although the role of metastasectomy remains unclear, emerging evidence suggests that surgical resection in carefully selected patients may confer a survival benefit [6].

Recent advances in chemotherapy and molecular-targeted therapies have contributed to improved systemic control and extended survival of patients with advanced pancreatobiliary carcinomas [8–11]. However, the role of aggressive local therapies including pulmonary metastasectomy remains controversial. Due to the rarity of these interventions, data on clinical outcomes and appropriate patient selection criteria are limited. Therefore, identifying prognostic factors associated with survival after pulmonary metastasectomy is critical for optimizing patient selection and guiding treatment strategies. The primary goal of pulmonary metastasectomy is curative resection, or, at a minimum, prolonging survival by removing all visible metastatic lesions. This procedure has become a standard therapeutic option for selected patients with lung metastases from various solid tumors such as colorectal cancer [12, 13]. Complete resection remains the most critical prognostic factor for the survival outcomes.

Although pancreatic carcinoma (PC) and biliary tract carcinoma (BTC) are clinically distinct entities with different disease courses and treatment strategies, opportunities to perform pulmonary metastasectomy for both malignancies are rare. Therefore, the aggregation of cases from both diseases using a nationwide multicenter database is necessary to enable meaningful analysis. Importantly, all major analyses were stratified by disease type to avoid drawing conclusions based solely on the pooled data. This study investigated the surgical outcomes and prognostic factors of pulmonary metastasectomy for pancreatobiliary carcinomas, using data from a nationwide registry in Japan. Our goal was to better define the clinical value of pulmonary resection in this rare setting and identify subgroups of patients who may benefit from surgical intervention.

## Methods

### Study design

This retrospective cohort study used data from the Japanese Association for Metastatic Lung Tumor (METAL-J) database between 1993 and 2019. All patients demonstrated sufficient pulmonary reserve and underwent preoperative evaluation, including chest and abdominal computed tomography (CT), to assess the primary tumor site and detect other potential metastatic lesions. Pathological diagnoses of pulmonary lesions were established at each participating institution based on histopathological evaluation, in conjunction with clinical history and radiological findings. Although centralized pathological reviews and detailed immunohistochemical data, such as thyroid transcription factor-1 (TTF-1) status, were not available, primary lung cancer was excluded based on comprehensive institutional diagnostic assessments. The histological subtype information of the primary malignancy was extracted from database records. However, a detailed histological classification was not consistently available across institutions, and a substantial proportion of cases were recorded as unknown or lacked sufficient detail to allow a reliable distinction between ductal adenocarcinoma and other histological subtypes. Due to these database limitations, only a subset of patients had available data: 35 patients were evaluated using fluorodeoxyglucose positron emission tomography (FDG-PET)/CT and 55 patients had serum cancer antigen 19 − 9 (CA19-9) levels measured prior to pulmonary metastasectomy. The threshold for the maximum standardized uptake value (SUVmax) was set at 2.5.

The criteria for pulmonary resection included: (1) controlled primary disease, (2) absence of active metastases at other sites, and (3) feasibility of complete resection of pulmonary metastases based on CT findings, regardless of the number of lesions. The presence of bilateral lung metastases was not considered to be a contraindication. During the postoperative follow-up, patients who developed additional pulmonary metastases were considered for repeat metastasectomy when feasible.

A retrospective chart review was conducted to collect the perioperative clinical data. The primary endpoints were overall survival (OS) and pulmonary metastasis-free survival (PmFS) after pulmonary metastasectomy. The disease-free interval (DFI) between surgery for primary tumors and pulmonary metastasectomies was also analyzed. A receiver operating characteristic curve analysis was performed to determine the optimal cut-off value for DFI. Based on this analysis, a DFI of 40 months was identified as the optimal threshold, with a sensitivity of 70.7% and specificity of 48.6%. This threshold was used to dichotomize patients for subsequent analyses.

All studies involving human participants were performed in accordance with the ethical standards of the institutional and national research committees as well as the 1964 Declaration of Helsinki and its subsequent amendments or other comparable ethical standards. This retrospective study was conducted with a waiver of authorization and was approved by the Institutional Review Board of Chiba University (approval number, 3628; approved on December 24, 2019). Approval was obtained from the ethics committee of each institution. Comprehensive informed consent was obtained from all participants.

### Statistical analysis

Categorical variables were compared using Fisher’s exact test. OS was defined as the time from the date of pulmonary metastasectomy to the date of death or last follow-up. Survival curves for OS and PmFS were generated using the Kaplan–Meier method and compared using the log-rank test.

Univariate analyses were performed using Cox proportional hazards regression models to identify factors associated with OS. We then constructed a multivariate Cox proportional hazards regression model using variables from univariate logistic regression analyses with a p-value of < 0.05. All analyses were performed using the EZR software program (Saitama Medical Center, Jichi Medical University, Saitama, Japan), a graphical user interface for R (R Foundation for Statistical Computing, Vienna, Austria) [14]. Given the limited sample size of the BTC subgroup, the number of variables included in the multivariate Cox proportional hazards model was intentionally restricted to minimize the risk of overfitting. Therefore, only variables that reached statistical significance in the univariate analysis and were considered clinically relevant were included in the multivariate model of the BTC subgroup.

## Results

### Patient demographics

The patient characteristics are summarized in Table 1. A total of 78 patients underwent pulmonary metastasectomy for pancreatobiliary carcinomas, including 56 patients with PC and 22 with BTC. Regarding pulmonary metastases, 52.6% were located in the right lung, 33.3% in the left lung, and 14.1% in both lungs. Among the 52 patients for whom data were available, the median CA19-9 level at pulmonary metastasectomy was 15.7 ng/mL (interquartile range [IQR], 6.7–50.8 ng/mL). Most patients (66.7%) had a single metastatic lesion, and mean maximum size of metastases was 1.8 cm (range, 0.5–6.7 cm). FDG-PET/CT data showed that 21 (26.9%) patients had PET-positive pulmonary lesions (SUVmax > 2.5), while 14 (17.9%) were negative. Data were unavailable for 43 (55.2%) patients. The median DFI from primary tumor resection to pulmonary metastasectomy was 36 months (IQR, 21–48 months): 37 months (IQR, 22–47 months) for patients with PC and 31 months (IQR, 16–48 months) for those with BTC. The clinicopathological characteristics of the primary pancreatobiliary carcinomas are summarized in Table 2.

**Table 1:** Patient characteristics of pulmonary metastasectomy for pancreatobiliary carcinomas

	All cases (*n*=78)		PC (*n*=56)		BTC (*n*=22)	
Age (years)						
Mean [Range]	67	[36–84]	67	[36–84]	67	[43–84]
Sex						
Male	41	53%	27	46%	14	64%
Female	37	47%	29	54%	8	36%
Lung metastasis side						
Right	41	53%	26	47%	15	68%
Left	26	33%	21	38%	5	23%
Bilateral	11	14%	9	16%	2	9%
CA19-9 at pulmonary metastasectomy [ng/mL] ^a^						
Median [IQR]	15.7 [6.7–50.8]		16.5 [9.2–53.2]		14.8 [6.5–44.0]	
SUVmax of pulmonary metastases on PET						
Positive	21	27%	14	25%	7	32%
Negative	14	18%	12	21%	2	9%
Unknown	43	55%	30	54%	13	59%
Operation						
Partial resection	52	67%	38	68%	14	64%
Segmentectomy	16	21%	9	16%	7	32%
Lobectomy	10	13%	9	16%	1	5%
Approach						
VATS	58	74%	42	75%	16	73%
Thoracotomy	20	26%	14	25%	6	27%
Number of metastases						
1	52	67%	36	64%	16	73%
2	15	19%	11	20%	4	18%
3	5	6%	4	7%	1	5%
≥4	6	8%	5	9%	1	5%
Maximum size of metastases						
Mean [Range] (cm)	1.8	[0.5–6.7]	1.8	[0.5–6.7]	1.8	[0.5–3.3]
0–1.0	18	23%	12	21%	6	27%
>1.0–2.0	37	47%	28	50%	9	41%
>2.0–3.0	15	19%	10	18%	5	23%
>3.0	8	10%	6	11%	2	9%
Lymphadenectomy						
ND0	51	65%	38	68%	13	59%
ND1	16	21%	12	21%	4	18%
ND2	11	14%	6	11%	5	23%
Lymph node metastasis						
Yes	4	5%	2	4%	2	9%
No	65	83%	48	86%	17	77%
Unknown	9	12%	6	11%	3	14%
Chemotherapy after pulmonary metastasectomy						
Yes	27	35%	21	38%	6	27%
No	14	18%	12	21%	2	9%
Unknown	37	47%	23	41%	14	64%
DFI (months)						
Median [IQR]	36 [21–48]		37 [22–47]		31 [16–48]	

PC, pancreatic carcinoma; BTC, biliary tract carcinoma; CA19-9, cancer antigen 19-9; SUVmax, maximum standardized uptake value; PET, positron emission tomography; IQR, interquartile range; VATS, video-assisted thoracoscopic surgery; ND, node dissection; DFI, disease-free interval

^a^ Data were available in 52 patients

**Table 2:** Characteristics of the patients with primary pancreatobiliary carcinomas

	PC (*n*=56)		BTC (*n*=22)	
Localization				
Pancreas body/tail	29	52%	-	
Pancreas head	27	48%	-	
Hilar bile duct	-		10	45%
Extrahepatic bile duct	-		12	55%
Histology				
Ductal adenocarcinoma	19	34%	7	32%
Other specified subtypes	4	7%	1	5%
Unknown / not specified	33	59%	14	63%
Lymph node metastasis				
Positive	21	37%	9	41%
Negative	24	43%	7	32%
Unknown	11	20%	6	27%
Distant metastasis				
Positive	3	5%	3	13%
Negative	40	72%	12	55%
Unknown	13	23%	7	32%
CA19-9 level at primary tumor resection [ng/mL] ^a^				
Median [IQR]	69.5 [19.5–186.7]		54.5 [10.4–209.0]	
SUVmax of the primary tumor on PET				
Positive	12	22%	2	9%
Negative	3	5%	1	5%
Unknown	41	73%	19	86%
Operation				
PD	38	68%	14	63%
Others	9	16%	7	32%
Unknown	9	16%	1	5%
Perioperative chemotherapy/radiation				
Neoadjuvant	4	7%	1	5%
Adjuvant	28	50%	9	41%
Unknown	24	43%	12	55%

Histological subtype information was extracted from the database records. “Ductal adenocarcinoma” includes tumors described as invasive ductal carcinoma or tubular adenocarcinoma. “Other specified subtypes” include non-ductal histology recorded in the database, such as intraductal papillary mucinous neoplasm–associated carcinoma, mucinous cystic adenocarcinoma, spindle cell sarcoma, and pseudosarcomatous carcinoma. Cases classified as “Unknown / not specified” indicate that histological subtype information was unavailable or lacked sufficient detail to allow reliable categorization

PC, pancreatic carcinoma; BTC, biliary tract carcinoma; CA19-9, cancer antigen 19-9; IQR, Interquartile range; SUVmax, maximum standardized uptake value; PET, positron emission tomography; PD, pancreaticoduodenectomy

^a^ Data was available in 46 patients

### Postoperative outcomes after metastasectomy

**Fig. 1:**
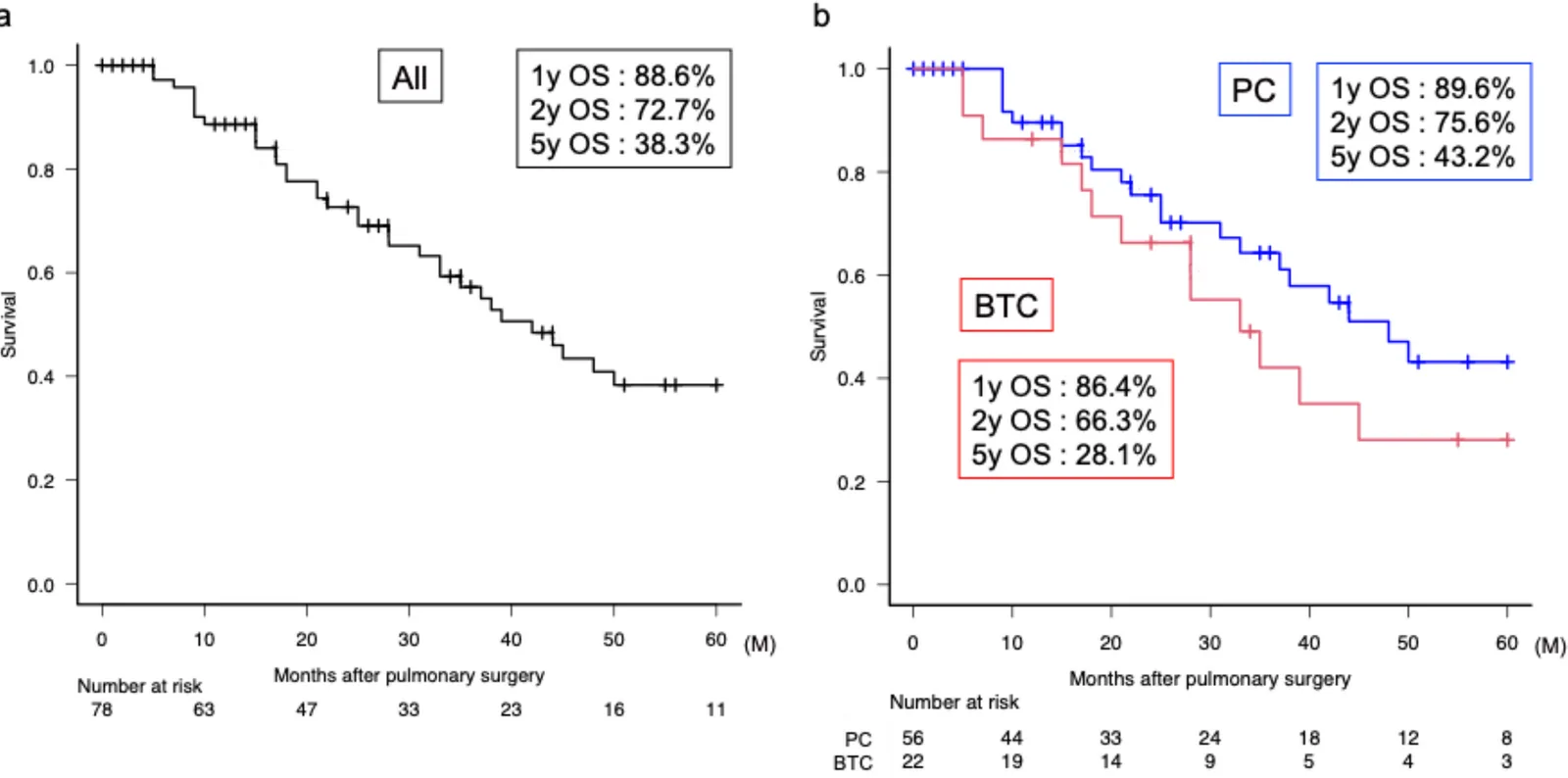
Overall survival after surgery for pulmonary metastases (**a**) Overall cohort (**b**) Subgroups of the pancreatobiliary group

**Fig. 2:**
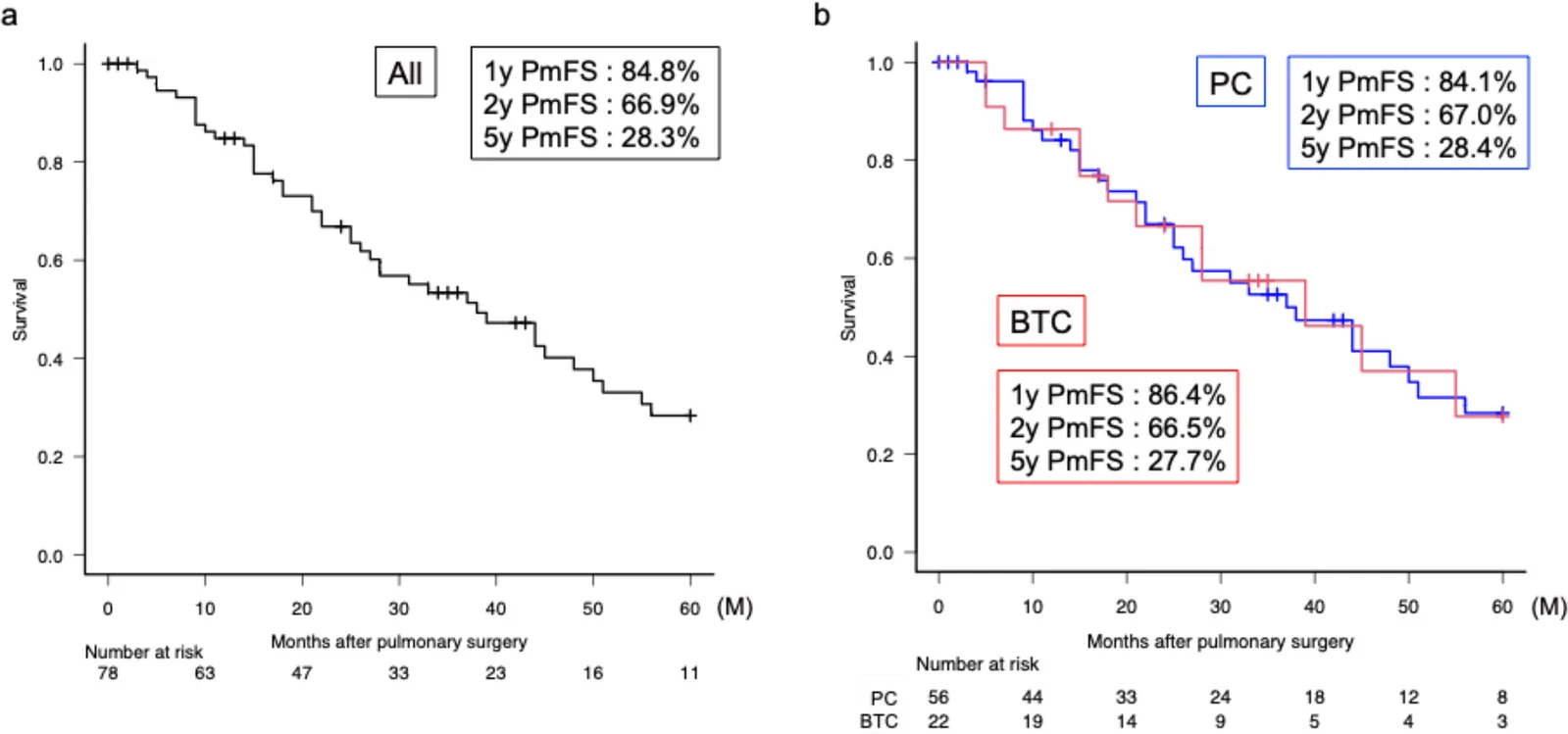
Pulmonary metastasis-free survival after pulmonary metastasectomy. (**a**) Pancreatobiliary all group; (**b**) Subgroups of the pancreatobiliary group

**Fig. 3:**
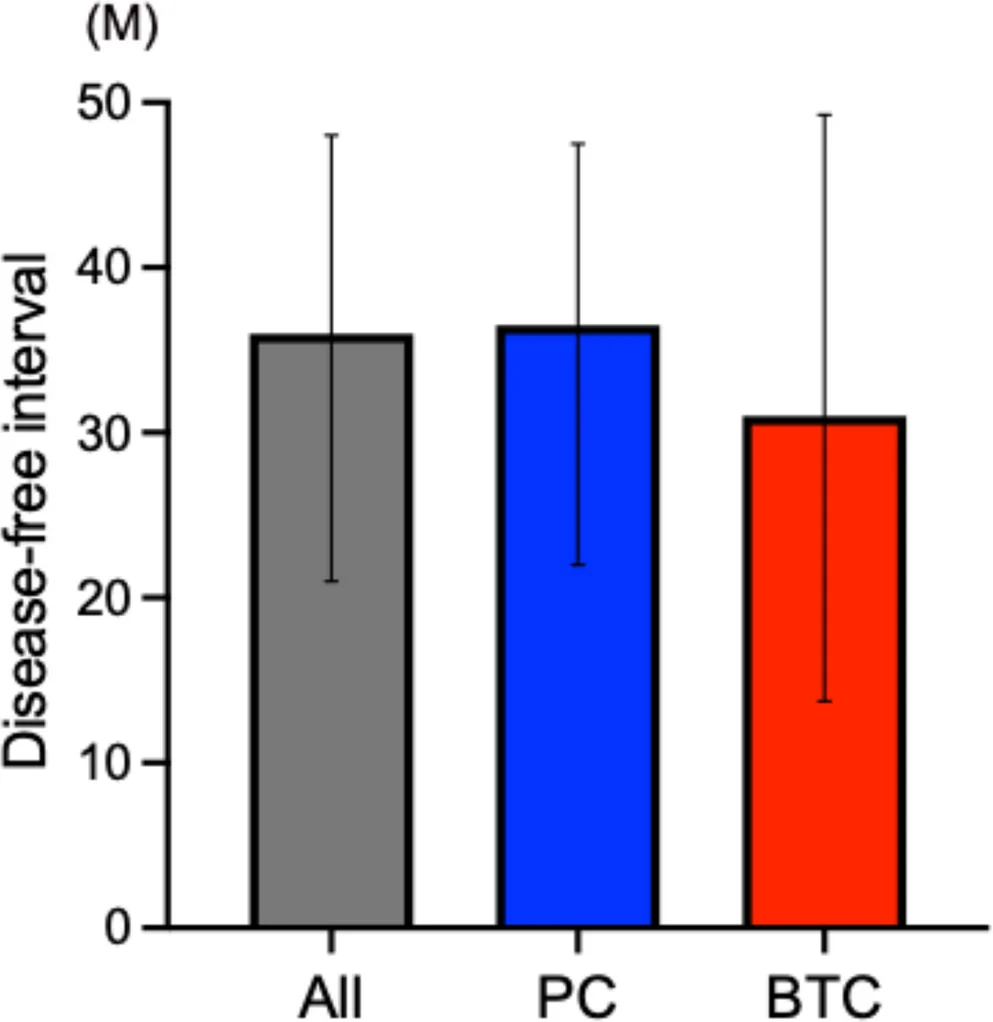
Disease-free interval between primary tumor resection and detection of pulmonary metastasis All, all the patients; PC, patients with pancreatic carcinoma; BTC, patients with biliary tract carcinoma

The one-, two-, and five-year OS rates after metastasectomy were 88.6%, 72.7%, and 38.3%, respectively (Fig. 1a). The OS rates of patients with PC and BTC after metastasectomy were 89.6% and 86.4% at one year, 75.6% and 66.3% at two years, and 43.2% and 28.1% at five years, respectively (Fig. 1b). The one-, two-, and five-year PmFS rates after metastasectomy were 84.8%, 66.9%, and 28.3%, respectively (Fig. 2a). The PmFS rates of patients with PC and BTC after metastasectomy were 84.1% and 86.4% at one year, 67.0% and 66.5% at two years, and 28.4% and 27.7% at five years, respectively (Fig. 2b). The median follow-up duration after pulmonary metastasectomy was 25 (IQR, 14–44) months. Median DFI of patients with pancreatobiliary carcinoma was 36.0 months (21.0–47.5, IQR), with 36.5 months (IQR: 22.0–46.5) in patients with PC patients and 31.0 months (IQR: 15.5–48.3) in those with BTC (Fig. 3).

### Predictors of mortality for patients with pulmonary metastasectomy for pancreatobiliary malignancies

**Fig. 4:**
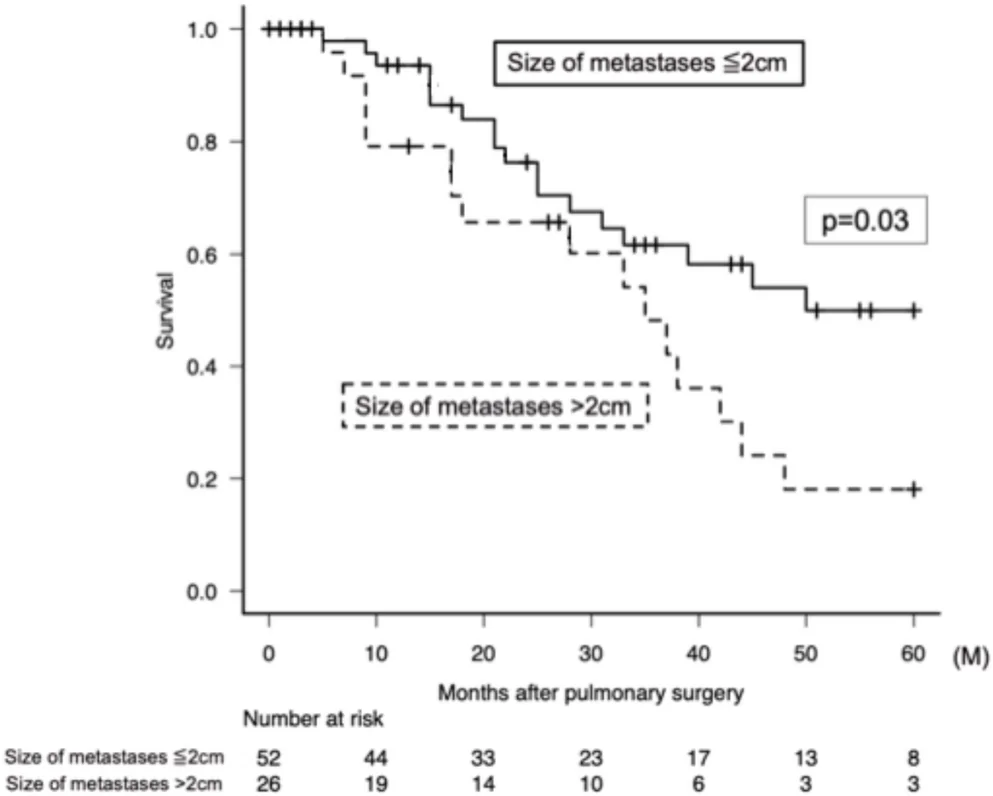
Overall survival after surgery for pulmonary metastasis with the size of the pulmonary metastases being > 2 cm or lower

A univariate analysis identified a tumor size of > 2 cm (hazard ratio [HR], 2.03; 95% confidence interval [CI], 1.06–3.90; *p* = 0.03) as a significant risk factor for a poor OS (Table 3; Fig. 4). In the cohort of patients with PC alone, no significant risk factors for a poor OS were identified, including bilateral pulmonary metastases (HR, 0.26; 95% CI, 0.06–1.11; *p* = 0.07). In patients with BTC specifically, both tumor size > 2 cm (HR, 4.01; 95% CI, 1.15–14.0; *p* = 0.029) and bilateral pulmonary metastases (HR, 5.40; 95% CI, 1.04–28.1; *p* = 0.045) were significantly associated with a worse prognosis. In an exploratory multivariate analysis, a tumor size of > 2 cm (HR, 19.54; 95% CI, 2.36–161.6; *p* = 0.006) and bilateral pulmonary metastases (HR, 8.10; 95% CI, 1.63–40.3; *p* = 0.001) were identified as factors associated with a poor OS among patients with BTC (Table 3). No other clinical variables were significantly associated with overall survival. Furthermore, of the 35 patients with available FDG-PET data prior to pulmonary metastasectomy, those with > 2.5 SUVmax had a poor prognosis compared with their counterparts (*p* = 0.02) (**Supplemental Fig. 1**). In a subset of 55 patients with recorded CA19-9 levels before pulmonary metastasectomy, no significant correlation was observed between the CA19-9 levels and prognosis (*P* = 0.12) (**Supplemental Fig. 2**).

**Table 3:** Univariate and multivariate analyses of the prognostic factors for survival after pulmonary metastasectomy for pancreatobiliary carcinomas with the Cox proportional hazards model

Variables	All patients (*n*=78)		PC (*n*=56)		BTC (*n*=22)			
	Univariate		Univariate		Univariate		Multivariate	
	HR (95% CI)	p-value	HR (95% CI)	p-value	HR (95% CI)	p-value	HR (95% CI)	p-value
Age ≧65	1.92 (0.92–4.01)	0.08	1.87 (0.69–5.10)	0.22	2.80 (0.88–8.92)	0.08	-	
Male	1.91 (0.96–3.79)	0.06	1.73 (0.74–4.07)	0.21	3.20 (0.84–12.3)	0.09	-	
Number of tumors resected ≧2	1.07 (0.55–2.09)	0.85	0.94 (0.41–2.19)	0.89	1.57 (0.48–5.14)	0.46	-	
Size of metastases >2 cm	2.03 (1.06–3.90)	0.03	1.39 (0.60–3.22)	0.44	4.01 (1.15–14.0)	0.029	19.5 (2.36-161.6)	0.006
Bilateral pulmonary metastases	0.64 (0.25–1.66)	0.36	0.26 (0.06–1.11)	0.07	5.40 (1.04–28.1)	0.045	8.10 (1.63-40.3)	0.001
Mode of pulmonary resection, wedge resection	1.32 (0.68–2.57)	0.41	0.94 (0.38–2.32)	0.9	1.45 (0.48–4.35)	0.51	-	
VATS	0.93 (0.45–1.91)	0.85	1.48 (0.54–4.02)	0.44	0.53 (0.17–1.62)	0.26	-	
Lymphadenectomy	1.32 (0.68–2.57)	0.41	0.79 (0.31–2.02)	0.62	1.82 (0.60–5.48)	0.29	-	
Lymph node metastasis	2.98 (0.90–9.91)	0.07	1.94 (0.26–14.7)	0.52	3.38 (0.70–16.4)	0.13	-	
DFI >40 months	1.31 (0.68–2.50)	0.42	1.35 (0.60–3.08)	0.47	1.11 (0.37–3.32)	0.85	-	
Perioperative chemotherapy/radiation for primary tumors	1.12 (0.58–2.20)	0.73	1.12 (0.47–2.66)	0.79	1.27 (0.42–3.80)	0.67	-	

PC, pancreatic carcinoma; BTC, biliary tract carcinoma; VATS, video-assisted thoracoscopic surgery; DFI, disease-free interval; HR, hazard ration; CI, confidence interval

## Discussion

Pancreatobiliary carcinomas are associated with a poor prognosis owing to their highly aggressive biological behavior. Even under optimal clinical trial conditions, the median survival time of patients with resected PC following adjuvant therapy ranges between 5.7 and 11.1 months [2]. PC is the leading cause of cancer-related mortality in both men and women, worldwide. Similarly, BTC has reported 5-year cumulative survival rates between 7 and 40%, although recent advances suggest a gradual improvement in the long-term outcomes [15, 16]. However, our multicenter retrospective analysis using data from METAL-J suggested that pulmonary metastasectomy may be a feasible treatment option in carefully selected patients, particularly in those with small, isolated metastases. It should be emphasized that the present study included only patients who were selected for pulmonary metastasectomy, thus representing a highly selected subgroup with favorable tumor biology and clinical characteristics. This inherent selection bias is unavoidable in retrospective surgical series and limits the generalizability of our findings. Therefore, the observed survival outcomes should not be interpreted as evidence supporting pulmonary metastasectomy for all patients with pulmonary metastases from pancreatobiliary carcinomas, but rather as outcomes achievable in carefully selected cases that meet strict surgical criteria.

The recurrence pattern of pulmonary metastases is of particular importance as it may significantly affect the prognosis of patients with PC. Previous reports have shown that patients with pulmonary-limited metastases from PC tend to have better outcomes than those with hepatic or peritoneal recurrence [17–22], and isolated pulmonary metastases from PC have been associated with a prolonged survival [21–23]. These findings may have important implications for surveillance strategies and therapeutic decision making. Advances in multi-omics analyses have suggested distinct immune microenvironments between pulmonary and hepatic metastases, with pulmonary metastases potentially maintaining a more robust immune response [24]. Furthermore, emerging data indicate that tumor-associated macrophages and NOD-like receptor protein 3 inflammasome activation play key roles in lung metastasis, suggesting that the lungs serve as an immunologically unique metastatic niche [25]. Recent studies have also shown that lung metastases are associated with increased tumor-infiltrating lymphocytes and elevated programmed death ligand 1 expression, suggesting that they may be more amenable to immunotherapeutic approaches using immune checkpoint inhibitors [26].

The potential benefits of pulmonary metastasectomy for isolated pulmonary metastases from pancreatobiliary carcinomas have been reported. Several retrospective studies and case series [5, 27–35] have demonstrated that surgical resection may contribute to long-term survival in carefully selected patients with PC. Notably, a nationwide analysis by Homma et al. showed a favorable prognosis following pulmonary resection in PC, supporting the utility of metastasectomy as a treatment strategy [21]. In the present study, the median survival after pulmonary metastasectomy was 24 months, which is consistent with previously reported ranges of 12–38.5 months [36]. Our results demonstrated surgical outcomes comparable to or superior to those of previous studies. Patients who undergo pulmonary metastasectomy for pancreatobiliary cancers are typically selected under rigorous criteria, including a thorough evaluation of extrathoracic disease and a comprehensive assessment of overall fitness. Patient selection based on the original Thomford principles [37] is essential to achieve favorable results. In our study, multidisciplinary evaluation emphasized the control of the primary tumor, absence of extrapulmonary metastases, and resectability, which likely contributed to the favorable outcomes.

Our analysis identified a tumor size > 2 cm as a significant predictor of a poor prognosis among patients with pancreatobiliary carcinomas. In the BTC cohort, bilateral pulmonary metastases were independent predictors of a poor survival. However, these findings should be cautiously interpreted. The multivariate analysis in the BTC subgroup was based on a small sample size, which likely contributed to the large hazard ratios and wide confidence intervals observed. Although the number of variables was restricted to reduce overfitting, limited statistical power precluded definitive conclusions. Therefore, these results should be regarded as exploratory and hypothesis-generating, rather than confirmatory. Notably, although tumor size was a prognostic factor for both PC and BTC with lung metastases, bilateral pulmonary metastases were only associated with worse outcomes for BTC. In contrast, no significant prognostic factors were identified in the PC group. These findings underscore the importance of careful patient selection when considering pulmonary metastasectomy for this population.

Several prognostic factors, including DFI [38], laterality [39], the number of pulmonary lesions [38, 39], and the serum CA19-9 levels [28, 38], have been reported in metastatic PCs. Interestingly, DFI, a commonly used selection criterion for metastasectomy, did not correlate with OS in our study. Although the median DFI was relatively long (36 months), suggesting indolent disease in this highly selected cohort, its lack of association with outcomes was notable. One possible explanation for this finding is that the prognostic impact of DFI may have been attenuated by some surgical selection bias. Patients with extremely short DFIs are unlikely to be referred for pulmonary metastasectomy, resulting in a relatively homogeneous cohort with indolent disease. Consequently, even patients with shorter DFIs in this study may have had favorable tumor biology that justified surgical intervention, thereby diminishing the discriminatory value of the DFI as a prognostic factor. Previous reports have suggested a potential association between the DFI and prognosis; however, other studies have found no such correlation [39], thus making it difficult to draw any definitive conclusions. Similarly, preoperative serum CA19-9 levels, which are often used to monitor the disease burden, did not predict the prognosis. This may reflect the limited sensitivity and specificity of CA19-9 in metastatic settings or the heterogeneity in tumor biology.

In addition to the established clinical parameters, metabolic activity measured using FDG PET/CT may provide additional prognostic value, as suggested by our limited cohort analysis. Specifically, SUVmax of pulmonary metastases has been proposed as a surrogate for tumor aggressiveness. Higher SUVmax values are correlated with increased proliferative potential and poor outcomes in various malignancies. Although the evidence remains limited for pancreatobiliary carcinomas [40, 41], our findings suggest a trend toward poorer prognosis in patients with high SUVmax pulmonary lesions, warranting further investigation. The incorporation of PET-based metabolic assessments into preoperative evaluations may enhance risk stratification and assist in refining the surgical indications.

This study is associated with several limitations. First, the analysis was based on data extracted from a database of patients who underwent pulmonary metastasectomy for metastatic tumors, representing a highly selected population. This selection bias is inherent to the retrospective and multi-institutional design and limits the generalizability of our findings. Second, the cases were collected over a period spanning more than two decades, during which systemic therapies, perioperative management, and diagnostic modalities for pancreatobiliary carcinomas have evolved substantially. Detailed information on chemotherapy regimens and treatment strategies was not available in the database. This temporal heterogeneity may have influenced the survival outcomes and should be considered when interpreting the results. Third, the heterogeneous and relatively short follow-up durations limit the assessment of long-term survival outcomes. Fourth, the inter-institutional variability in PET imaging due to differences in scanner models and protocols was not adjusted for. As the data were collected retrospectively from multiple centers, the SUV measurements may have been affected by technical differences. Future studies should consider standardization methods to minimize variability. Another important limitation of this study was the incomplete availability of detailed histological subtype information for primary tumors. Although histological data were recorded in the database, many cases were classified as unknown or lacked sufficient detail to permit a reliable subtype-based analysis. Consequently, the potential differences in the tumor biology and prognosis among distinct histological subtypes could not be fully evaluated, which may affect the interpretation and generalizability of our findings. Furthermore, detailed information regarding the clinical course prior to pulmonary metastasectomy, including whether pulmonary metastases represented the first site of recurrence or occurred after prior recurrence at other sites, was not consistently available. This information is clinically important for surgical decision making and represents another limitation of the present study. Moreover, subgroup analyses, particularly for BTC, were constrained by the small number of patients, which may have resulted in statistical instability and limited the robustness of the multivariate modeling. Finally, the sample size was relatively small. Prospective multicenter studies with larger cohorts and standardized follow-up protocols are warranted to validate the role of pulmonary metastasectomy in this population. The continued accumulation of cases and longer observation periods are essential to clarify the true indications and prognostic effects of pulmonary metastasectomy in patients with pancreatobiliary carcinomas.

In conclusion, pulmonary metastasectomy may be an appropriate therapeutic option for carefully selected patients with pancreatobiliary carcinoma. Based on our findings, patients with limited metastatic burden, smaller tumor size, unilateral disease, and feasibility of complete resection in the absence of uncontrolled extrapulmonary metastases may represent a subgroup with favorable outcomes. These factors should be regarded as suggestive rather than prescriptive, and require validation in larger prospective studies. Multicenter prospective clinical trials involving a sufficient number of patients are therefore required to elucidate the significance of surgical treatment in patients with pulmonary metastases from pancreatobiliary carcinoma.

## Supplementary Information

Below is the link to the electronic supplementary material.

Supplementary Material 1

## Data Availability

The datasets analyzed in this study are available from the corresponding author upon request. De-identified clinical data and statistical analysis codes can be shared for academic and noncommercial purposes. Data sharing is subject to institutional and ethical regulation.
