# Supplementary material for: Survival after pulmonary metastasectomy for pancreatobiliary carcinomas: insights from a nationwide multicenter database
Source: Surg Today. 2026 Jul 20;56(11):2772–81. doi: 10.1007/s00595-026-03389-8 (PMC13722726; doi:10.1007/s00595-026-03389-8)
Supplement: Supplementary file 1 [file 595_2026_3389_MOESM1_ESM.docx]

**Supplemental Figure 1.** Overall survival after surgery for pulmonary metastasis with >2.5 of fluorodeoxyglucose-positron, emission tomography (PET)/CT maximal standardized uptake value (SUVmax) or lower. (n=35)

**Supplemental Figure 2.** Overall survival after surgery for pulmonary metastasis and the serum cancer antigen 19-9 (CA19-9) level (n=55)
